# The Feed Factor: Estrogenic Variability in Lab Animal Diets

**DOI:** 10.1289/ehp.114-a640

**Published:** 2006-11

**Authors:** M. Nathaniel Mead

Animal studies have long been a cornerstone of biomedical and environmental health research, and scientists need assurance that animals used in these studies are being cared for in ways that will not unknowingly influence experimental outcomes. But a growing number of scientists have voiced concern over the possibility that certain estrogenic compounds present in lab animal feed may skew test results. These compounds are deemed potentially problematic because they can bind to estrogen receptors and induce estrogen-like effects in animals, humans, and cells grown in culture. Some experts have advocated strict standardization of rodent chows and even the removal of dietary phytoestrogens.

This emergent controversy was the focus of “DIET II—The Effect of Variability in Estrogenic Activity of Commercial Animal Feeds: Interaction with Manufacturers, NIH Officials, and Scientific Societies to Develop a Solution,” a full-day meeting held 3 August 2006 in Research Triangle Park, North Carolina. The meeting, the second in a series on the topic, was cosponsored by the NIEHS and the NIH Office of Dietary Supplements (ODS). Discussions at the meeting centered on the variation in estrogenic activity between feed batches, the effects of these estrogenic components on endocrine-related end points, and the difficulties inherent in comparing, interpreting, and reproducing these end points over time within and between different laboratories when background levels of diet-related estrogenic activity are not adequately documented. Findings presented at this meeting made it clear that researchers studying estrogen-related end points can not afford to overlook the influence of the test rodent’s diet.

“This workshop is an excellent example of the cumulative and self-correcting nature of the scientific process,” said ODS nutritionist Elizabeth Yetley, a conference co-organizer. “That is, through the accumulation of results from a body of experimental evidence, the importance of approaches for better-defined animal diets relative to their potential estrogenic activity have been identified, and measures to improve future research in this area are being undertaken by the scientific community.”

Participants at the conference—organized by NIEHS scientists Jerrold Heindel and Julius Thigpen, along with Yetley and University of Missouri–Columbia biologist Frederick vom Saal—included investigators from the endocrine disruptor research community as well as representatives from animal feed companies. This spectrum of representatives reflected the fact that, in Heindel’s words, “researchers and animal care divisions of research institutions are beginning to pay attention to the phytoestrogen issue, and feed manufacturers want to know what the scientific community wants.”

Heindel described a sincere interest on the part of both sides to reach a “win–win” solution, one that would yield animal diets of a known estrogenicity that could be used by researchers in all fields of physiology and toxicology, but that also would not unduly burden the feed manufacturers.

## The Chow Variable

The idea that a dietary background of phytoestrogens may modulate some responses to environmental estrogens in rodent studies is not new. In the early 1980s, NIEHS investigators consulted the institute’s Quality Assurance Laboratory (QAL), headed by Thigpen, in an effort to determine why scientists were unable to duplicate data from outside laboratories—or vice versa—and why sometimes they couldn’t even duplicate their own data. This led to the awareness that animals were being fed vastly different diets not only in different labs but also within the same lab.

In the October 1987 issue of *Laboratory Animal Science*, Thigpen and colleagues reported for the first time that commercially available rodent diets differed considerably in estrogenic activity. Their conclusion: the composition of the animal feed was important when performing or comparing results from studies involving estrogenic compounds. At the time, they proposed that a standardized diet with minimal estrogenic activity would be desirable for bioassays on estrogenic substances.

Recent studies in the United States and Europe have confirmed that the variability in estrogenic activity in rodent diets is primarily due to the changing phytoestrogen content—often the proportion of soy isoflavones, although alfalfa and brewer’s yeast also contribute to this effect. There appears to be tremendous variation in isoflavone levels between batches of the same feed.

“We now know that the phytoestrogen content can vary three- to sixfold between different batches of the same diet, producing significant differences in [developmental events] in mice,” says Thigpen. “Researchers need to be concerned about the background influence of these dietary estrogens, particularly in studies of estrogen-sensitive signaling pathways and of the impact of endocrine-disrupting chemicals on reproduction and reproductive physiology.”

To underscore the fundamental nature of the debate, Thigpen emphasized at the meeting that the diet selected for any controlled study should reduce variables, not increase them. For example, several studies—including one published in the May 2001 issue of *Laboratory Investigation*, have shown that soy isoflavones provided in standard rodent chow actually diminished the uterine effects of pharmaceutical and industrial estrogens in ovariectomized rats. There are also examples, says Heindel, where the positive estrogenic effect of diethylstilbestrol was not detected in experiments due to the high level of phytoestrogens in the diet.

## Working Around Phytoestrogens

Unfortunately, though manufacturers can control how much soy protein goes into feeds, they have no control over the isoflavone content of the soybeans themselves. Variations in the isoflavone content relate primarily to variations in climate, timing of harvest, and storage. “Our scientists have shown that there’s up to a tenfold variation in the phytoestrogen content of soybeans, and this is largely a function of increased temperature, increased water stress, and carbon dioxide in the [soy] growing atmosphere,” says David Klurfeld, a nutrition scientist with the USDA Agricultural Research Service. He notes that the recent heat wave on the East Coast will likely impact the phytoestrogen content of soybeans, so that “next year we’ll unknowingly be using soy-based feeds and soy products that have a higher phytoestrogen content.”

Kenneth Setchell, an isoflavone researcher and pediatrics professor at the University of Cincinnati College of Medicine who coauthored the 2001 *Laboratory Investigation* paper, is disturbed by what he perceives as a pervasive lack of awareness concerning this issue. “Very few researchers have a clue as to what diet is being fed to their animals,” Setchell said. Besides the fact that these diets can have a profound impact on physiology and thus on experimental outcomes, when it comes to isoflavone consumption rates and metabolic capacities, many key differences exist between mice and rats, between different strains of the same species, and between rodents and humans. “These differences could potentially make it very difficult to compare findings from different studies of the same issue,” Setchell said.

Compared to humans, for example, rodents consume far more isoflavones per unit of body weight and produce much more S-equol (relative to R-equol, which is higher in humans), a phytoestrogen metabolite that closely resembles estradiol. It also has been demonstrated that Sprague-Dawley rats are much less sensitive than other lab rat species to the effects of phytoestrogens.

One suggestion at the meeting was that estrogenic activity of feeds be reported in every scientific paper that has an endocrine component. But Setchell pointed out the potential for phytoestrogens affecting nonendocrine end points as well. He recounted the story of a biology professor who was studying a rodent gene knock-out model for cardiomyopathy. This particular model typically succumbs to congestive heart failure in the first days of life. “[The investigator] noticed that animals fed a standard Purina animal chow were not dying on schedule,” said Setchell. “Evidently something in the Purina chow was curbing the development of cardiomyopathy.” That something turned out to be phytoestrogens, and specifically the isoflavones from soy protein.

A similar argument for reporting estrogenic activity of animal feeds can be made in the context of studying neurobehavioral disorders, as dietary phytoestrogens may influence these outcomes as well, with both high and low levels associated with adverse effects in animal models. A study in the January–February 2002 issue of *Neurotoxicology and Teratology* showed that the soy phytoestrogen content of the diet exerts specific influences on learning, memory, and anxiety-related behaviors. In one set of experiments, for example, researchers found that adult rats fed a “phyto-rich diet” had more anxiolytic effects than animals fed a “phyto-free diet.”

Data presented by vom Saal indicated that even diets thought to be devoid of phytoestrogens—such as diets based on casein, a milk protein—may contain significant amounts of estrogenic activity and that that estrogenic activity can vary up to sixfold from batch to batch. Thus, even switching to a casein-based diet would not prevent the problem of variable estrogenicity of animal diets.

The key, vom Saal argued, is to try to find a feed that provides the optimal level of serum estradiol during and after pregnancy—this could mean different diets for these different life stages. “We believe that there are animal diets that allow us to study rodents in ways that are highly predictive of human health outcomes,” he said; for example, one such diet consists of a typical soy-containing feed that is roughly comparable to what a mouse foraging in a natural environment might consume. “On the other hand,” he said, “some [rodent] diets can change the animal so profoundly that it’s unusable for those experiments.”

Retha Newbold, a developmental reproductive biologist at the NIEHS Laboratory of Molecular Toxicology, has investigated the effects of phytoestrogens and endocrine-disrupting chemicals for years. She noted, “There are some experimental animals that simply thrive and reproduce better on diets with phytoestrogens.” Further, she added, it should be up to the investigators themselves to determine if their particular experiment calls for a phytoestrogen-free diet or not, but certainly researchers need to know the phytoestrogen content and the hormonal activity of the feed they are using.

## Recommendations and Agreements

At this time, it may be impossible to determine to what extent phytoestrogens in animal diets have affected the results of previous research. Nonetheless, no one at the workshop would disagree that the concern is real, given what is now known about these compounds’ substantial variability, potency, and ability to significantly alter molecular end points and gene expression.

Scientists at the meeting agreed that the background estrogenic activity effects of animal feeds are important and need to be controlled. They also agreed that there can not be one optimal diet for all research. Deciding on a standard rodent diet will likely depend on the specific objectives of the study and on the end points being evaluated. There was also consensus that many animal researchers are not aware of the problems posed by not knowing the hormonal activity of their diets and how this activity can vary from batch to batch.

Participants further agreed that simply stating there are no known sources of estrogenicity in diets is not sufficient; total estrogenicity of diets needs to be measured. They also agreed to work with feed manufacturers so that researchers could have access to diets with known estrogenicity. This would not mean that manufacturers would need to produce new diets, but only that they test diets for total estrogenicity including the presence of phytoestrogens, mycotoxins, zearalenone, and any other ingredient that might cause an estrogenic or hormonal response in animals.

The actual details of the testing and reporting of the results were not completely worked out, but participants concluded that the best solution would be to perform assays for known hormonally active chemicals and bioassays that could identify hormonal activity of unknown sources. Future research will be needed to determine the range of estrogenic activity for a particular type of feed that would be acceptable and that would not significantly alter the lab animal’s phenotype or interfere with responses to exogenously administered estrogens in bioassays.

“The NIEHS workshop sought to address a very complex and challenging methodological issue involved in the study of one type of bioactive substances found in foods and dietary ingredients,” said Yetley. “As such, the findings from this workshop will not only help to improve the quality of future research related to phytoestrogens, but will also help inform us of better ways to deal more generally with similar methodological challenges with other botanical and food-derived substances.”

## Figures and Tables

**Figure:**
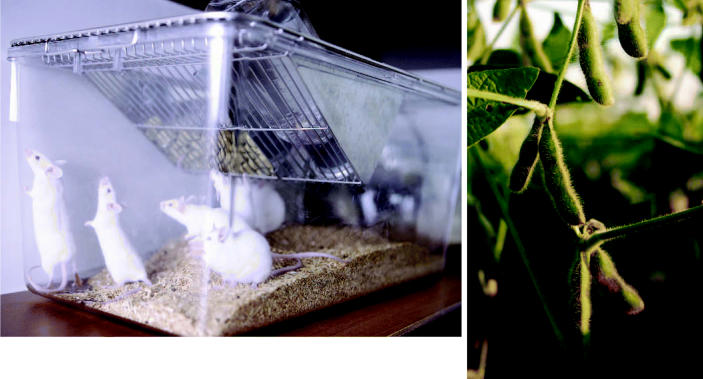
It’s in there Scientists and lab animal feed makers recently met to discuss problems resulting from unwanted effects of phytoestrogenic components such as soy in animal diets.

